# Large Mammalian Animal Models of Heart Disease

**DOI:** 10.3390/jcdd3040030

**Published:** 2016-10-05

**Authors:** Paula Camacho, Huimin Fan, Zhongmin Liu, Jia-Qiang He

**Affiliations:** 1 Department of Biomedical Sciences and Pathobiology, College of Veterinary Medicine, Virginia Tech, Blacksburg, VA 24061, USA; pcamacho@vt.edu; 2 Research Institute of Heart Failure, Shanghai East Hospital of Tongji University, Shanghai 200120, China; frankfan@tongji.edu.cn (H.F.); liu.zhongmin@tongji.edu.cn (Z.L.)

**Keywords:** dog, sheep, pig, non-human primates, heart disease model

## Abstract

Due to the biological complexity of the cardiovascular system, the animal model is an urgent pre-clinical need to advance our knowledge of cardiovascular disease and to explore new drugs to repair the damaged heart. Ideally, a model system should be inexpensive, easily manipulated, reproducible, a biological representative of human disease, and ethically sound. Although a larger animal model is more expensive and difficult to manipulate, its genetic, structural, functional, and even disease similarities to humans make it an ideal model to first consider. This review presents the commonly-used large animals—dog, sheep, pig, and non-human primates—while the less-used other large animals—cows, horses—are excluded. The review attempts to introduce unique points for each species regarding its biological property, degrees of susceptibility to develop certain types of heart diseases, and methodology of induced conditions. For example, dogs barely develop myocardial infarction, while dilated cardiomyopathy is developed quite often. Based on the similarities of each species to the human, the model selection may first consider non-human primates—pig, sheep, then dog—but it also depends on other factors, for example, purposes, funding, ethics, and policy. We hope this review can serve as a basic outline of large animal models for cardiovascular researchers and clinicians.

## 1. Introduction

Cardiovascular disease is considered the major cause of morbidity and mortality worldwide. Over the past several years, significant achievements have been made in the management of cardiovascular disease that depended on the use of experimental animal models: a small (e.g., mice or rats) or large (e.g., dogs or sheep) animal model [1,2,3]. For research aimed at clinical translation, it is imperative that initial results from small rodent studies be confirmed in a large animal model that more closely resembles humans with the high percentage of genetic conservation [4,5,6]. Determining the best experimental model to be used requires a number of decisions and compromises so as to obtain the optimal balance between the quantity and quality of data produced and the relevance of the data to the condition under investigation. The nature of the question under study greatly influences the selection of the most appropriate investigative models and endpoints of cardiac function and malfunction [7]. It is important to standardize the procedures used, in order to obtain relevant and reproducible results that can be compared with other findings.

Canines and swine have been the most frequently used species, with primates typically excluded due to cost, and a vigorous debate has ensued over which animal most closely resembles the human [7]. Important anatomical and functional differences between dogs and pigs should be considered before use. For instance, the coronary circulation of pigs has no anastomoses between vascular branches, whereas the coronary circulation of dogs can be extensively collateralized [8]. In general, it is thought that the young human heart may be somewhat “pig-like” whereas the older heart with ischemic heart disease (which promotes collateral growth) may be more “dog-like” [7].

No ideal animal model of the human cardiovascular system exists and studies should not rely on a single animal model to address all questions. Nevertheless, differences between laboratory animals and humans are decreased as the body/heart weight of the model approaches that of humans [9]. The main advantages of in vivo studies using large mammalian hearts are that: (1) they can legitimately claim to be the most physiologically and/or clinically relevant; (2) they allow chronic studies to be undertaken; (3) they allow cardiac functions and responses to be assessed in the intact animal [7]; and (4) they are probably the best model for new drug screening and toxicity tests due to their conserved molecular mechanisms to humans rather than to small animals such as rodents [4,5].

We must remember, however, that these animal models are far from ischemic heart disease present in humans. Most animal studies involve the sudden occlusion of a coronary artery in what was previously healthy tissue. This differs greatly from the complex and progressive development of human cardiovascular disease with its underlying vascular perturbations and genetic and environmental components [7]. There are also economic and logistic disadvantages to using large animal models. Most notably, large animal models are much more expensive to purchase and maintain in animal facilities than small animal models; the daily housing fees of large animals are 30 to 90 times more expensive than those of mice [9].

## 2. Dogs

Canine and human hearts share many characteristics on both the organ and cellular levels. Canine heart rate, body weight, and heart weight are more comparable to humans than the heart rate, body weight, and heart weight of mice, rats, and rabbits. Due to their size, almost all in vivo techniques used to assess contractility of human hearts can be utilized in canines. Housing and maintenance of canine models is considerably more expensive than small rodents and cats; therefore, this issue must be taken into consideration when long-term studies and disease models are being considered. One disadvantage, unique to canines, is obtaining the necessary approval for performing experiments in this species [9].

### 2.1. Blockage-Induced Myocardial Infarction (MI)

To induce MI in dogs, the chest is opened under general anesthesia via the left, fourth intercostal space, the pericardium is opened to expose the heart, and the proximal left anterior descending (LAD) coronary artery is ligated below the first diagonal branch [10,11]. Care needs to be taken to ligate all visible epicardial collaterals [12]. As in mice, MI significantly promotes cell proliferation in the canine epicardial region, thus substantially increasing the thickness of this region in an infarcted heart. Data indicate that MI reactivates a fetal program, including increased proliferation and upregulation of fetal genes [13].

Canine atrial MI is considered unusual because it is rarely diagnosed, but it often goes undetected. Patients with atrial MI have increased supraventricular arrhythmia risk. In an experiment using 40 dogs, isolated right atrial (RA) MI was generated by double ligation of the right intermediate atrial artery. MI dogs had substantially increased spontaneous ectopic activity, which manifested as premature atrial complexes and atrial tachyarrhythmias. Chronic coronary artery occlusion created substrates for both spontaneous atrial ectopy and prolonged atrial fibrillation in dogs [14].

#### 2.1.1. Chronic Ischemic Heart Failure (HF)

This new model combines a chronic infarct matrix with ongoing small vessel coronary disease to produce moderate levels of left ventricle (LV) dysfunction. In mongrel dogs, MI could be induced by ligation of their LAD coronary artery. Using a diagnostic coronary catheter, the left main coronary artery was engaged under fluoroscopic guidance. The left circumflex coronary artery was identified and sonicated latex beads were slowly injected and flushed into it. The procedure was repeated weekly until the target LV ejection fraction (LVEF) was reached [15]. This procedure reproduced chronic ischemic HF.

#### 2.1.2. Sudden Cardiac Death (SCD)

The anatomic and functional changes induced by MI or atrioventricular (AV) block may serve as the substrates for SCD [16]. However, the presence of MI or AV block alone does not lead to a high incidence of SCD. A combination of spontaneous ventricular arrhythmia (trigger) and the underlying anatomic and electrophysiological abnormalities (substrate) account for a high incidence of spontaneous ventricular tachycardia (VT), fibrillation, and SCD [10]. To produce an animal model of SCD, an ablation catheter is inserted via the right femoral vein to the AV nodal region under fluoroscopy guidance. Then, radiofrequency energy was applied to create a complete AV block and an implantable cardioverter-defibrillator was used to monitor electrical activity. Later, the LAD was ligated below the first diagonal branch to create anterior wall MI and the dogs were monitored constantly until SCD occurred [17].

### 2.2. Spontaneous Arrhythmogenic Right Ventricular Cardiomyopathy (ARVC)

ARVC is a primary familial heart muscle disease associated with substantial cardiovascular morbidity and risk of SCD. Boxer dogs are a spontaneous animal model of ARVC likely coming with SCD. In addition to sudden death, this canine model is characterized by VT of suspected right ventricle (RV) origin and structural RV abnormalities. The combined clinical profile and pathological abnormalities closely resemble those of the human disease [18]. Animals are clinically diagnosed with ARVC by echocardiography, electrocardiography, and 24-h ambulatory electrocardiographic (Holter) monitoring [19].

In Boxers, chromosome 17 was identified as the most significant region associated with the disease phenotype. Dogs homozygous for this variant were all affected, and on average were more severely affected than dogs heterozygous for the variant [20]. The disease in Boxers is associated with a significant remodeling of the structures involved in cell–cell communication. Loss of gap junctions may represent a substrate in the development of ARVC-related ventricular arrhythmias [19].

### 2.3. Non-Ischemic Heart Failure (HF)

To produce non-ischemic heart failure, ventricular dysfunction is induced by RV tachypacing (TP). Briefly, adult dogs are instrumented with pacemakers with the lead placed in the RV apex. TP is performed at 180 beats per minute (bpm) for 2 weeks, 200 bpm for 6 weeks, 180 bpm for 2 months, 160 bpm for 6 months, and then 120 bpm thereafter [21]. This models the process of arrhythmogenesis during heart failure progression.

### 2.4. Duchenne Muscle Dystrophy (DMD)

Among the various animal models of DMD, the Golden Retriever muscular dystrophy (GRMD) dog is considered the best model in terms of size and pathology onset. The GRMD dog carries a point mutation in the X-linked dystrophin gene and has a clinical course very similar to that of human patients, characterized by progressive muscle wasting, degeneration, fibrosis, and a shortened lifespan. Cardiac involvement in GRMD dogs has been demonstrated by electrocardiographic studies, with the onset of a progressive cardiomyopathy similar to DMD patients [22].

### 2.5. Chronic Valvular Disease

This disease, which most often affects the mitral valve, is similar to mitral valve prolapse in humans. However, it has a much more progressive nature, often leading to chronic HF. This disease occurs primarily in middle-aged to older small- and medium-sized dogs and is particularly common in certain breeds, such as the Cavalier King Charles Spaniel, Dachshunds, miniature and toy Poodles, Chihuahuas, and Terrier breeds [23]. Research for this common disease in dogs can be translated to human valvular disease.

### 2.6. Dilated Cardiomyopathy (DCM)

The second most common heart disease in dogs is DCM. Giant dog breeds, such as Irish Wolfhounds, Great Danes, Saint Bernards, and Newfoundlands, are at risk. Doberman Pinschers and Boxers are also highly predisposed [23]. To serve as models, these animals are instrumented with implanted probes and monitored for weeks or months during the slow development of heart failure, possibly by taking hemodynamic measurements in the conscious state. This has the advantage of producing a physiologically and/or clinically relevant model that allows assessments of cardiac function in the intact animal [8].

This model has provided clues as to the possible pathological mechanisms that involve DCM. One of the most commonly reported canine breeds with familial DCM is the Doberman Pinscher. A deletion in the PDK4 gene is associated with the development of the disease. This gene plays a pivotal role in regulating the energy metabolism and its damage results in a chronic energy starved state [24]. During progression of the disease, cardiac arrhythmia is a common problem but the type of arrhythmia varies. Ventricular arrhythmias predominate in the Doberman Pinscher and Boxer (SCD is very common in these two breeds), whereas atrial fibrillation (AF) is more common in Irish Wolfhounds, Newfoundlands, and other giant breeds. Atrial premature contractions are the primary arrhythmia found in Cocker Spaniels with DCM. DCM typically results in severe dilation of all four cardiac chambers with modest thinning of the ventricular free walls and septum [23].

## 3. Sheep

Sheep, as a large animal model with its similarities to the human heart, are approved to serve as a good pre-clinical animal model for cardiovascular research [9]. At cellular and molecular levels, the predominate (~100%) myosin heavy chain isoform in the sheep heart is the slow β-MHC similar to humans (~95%). In terms of functionality, the contractile and relaxation kinetics of sheep cardiomyocytes are also similar to human heart cells and both sheep and human hearts have a positive force–frequency relationship. In addition, the resting heart rate in sheep (60–120 bpm) is similar to humans (60–75 bpm) and so are the systolic (~90–115 mmHg) and diastolic (~100 mmHg) pressure in sheep to human (120 mmHg and 80 mmHg, respectively). Several sheep models of heart disease have been reported [9,25].

### 3.1. Ligation-Induced Myocardial Infarction in Fetal Sheep

With the exception of gene-modified murine models, the availability of suitable animal models to assess human stem cell fate and bio-distribution is very limited. As most available animal models are associated with the necessity for immunosuppressive therapy when applying human cells, the clinical relevance of findings obtained from such animal models is compromised. In contrast to other animal models either being genetically modified or requiring immunosuppressive therapy for the assessment of human stem cells, the fetal sheep has a normal functioning immune system but is still able to support cell engraftment and survival if the cells are transplanted before day 75 of gestation (term 145 days). The fetal sheep represents a highly interesting animal model to study human cell-fate, offering experimental opportunities that are not available in murine models [26].

After the ewes are placed in dorsal recumbency on the operating table, the uterus is exteriorized through a maternal midline laparotomy. Following digital palpation of the fetus, the uterus is opened through a 10 cm incision. The amniotic fluid is collected, stored in a sterile reservoir and put back into the ewe’s belly to maintain temperature until the end of the procedure. While the upper part of the fetal body remains within the maternal uterine cavity, the fetal chest is opened via a left-side mini-thoracotomy (fourth intercostal space). After sharp dissection of the pericardium, the heart is positioned for optimal access of the anterior wall and apex. To achieve a sufficient MI involving the anterior wall and apex, the LAD (six appropriate diagonal branches) are suture ligated. Sufficient ligation is confirmed by instant changes in the movement of the regional wall and the color of the anterior-apical area. The fetal chest is closed and carefully re-positioned with particular regard to the umbilical cord. The amniotic fluid is re-injected or replaced with sterile normal saline plus penicillin-G before closing the uterus [26,27,28,29].

There is a marked difference between fetal and adult responses to MI. Adults form a scar by replacing myocardium with collagen, and show infarct expansion and decline in cardiac function. The fetus, on the other hand, demonstrates a capability of healing by myocardial regeneration, which is associated with minimal cellular inflammatory response and cellular proliferation both in the infarct and border zone [28]. Fetal cardiac regeneration after MI is due to increased recruitment and differentiation of cardiac progenitor cells in the infarct region [27] and a low cellular inflammatory response [28]. After MI, fetal hearts present decreased expression of genes involved in the regulation of inflammation, extracellular matrix remodeling, apoptosis, cell cycle, cell migration and proliferation, and response to wounding compared with adult hearts [29]. This model of regenerative cardiac healing can be used to identify factors that are important in promoting the regenerative phenotype.

### 3.2. Embolization-Induced MI

Embolization MI could be induced by cardiac catheter through the femoral or carotid arteries. Briefly, introducer sheaths are inserted, and under fluoroscopic guidance, a coronary guiding catheter is advanced into the aortic root. An intracoronary guidewire is then positioned in either the LAD or circumflex artery, depending on the site of the planned MI. An infusion catheter is advanced over the wire, which was subsequently removed. A mixture, comprising sterile Gelfoam suspended in saline, is injected via the infusion catheter and flushed through with contrast medium. MI is confirmed by elevation of S- and T-wave (ST) segment on Electrocardiogram (ECG) monitoring and the appearance of new LV segmental abnormalities on echocardiography. The procedure can be repeated until the target LVEF is reached [30].

### 3.3. Sudden Cardiac Death (SCD)

Chronically telemetered sheep with bradycardia and chronic MI can serve as a model of spontaneous sustained VT and SCD in the absence of acute ischemia or direct stimulation of the autonomic nervous system, in hearts that are almost as large as adult human hearts. The incidence of spontaneous sustained VT was 22 times higher in the presence of both chronic MI and bradycardia compared to bradycardia alone. To generate this model, a transvenous defibrillation catheter is placed via the left external jugular vein into the RV. The lead is connected to both an implantable cardioverter defibrillator (ICD) and an implantable telemetry unit. This catheter is used for back-up pacing, internal defibrillation, and continuous recording of the RV electrogram. A second catheter is placed via the coronary sinus into the great cardiac vein over the anterior wall of the LV to record an electrogram. An ablation catheter is inserted via the external jugular vein toward the RA. Radiofrequency energy delivery is continued until complete heart block is produced as indicated by AV asynchrony and ventricular bradycardia. Finally, infarction is induced by angioplasty balloon occlusion of the left anterior descending coronary artery distal to the second diagonal branch for 150 min. Post-ablation pacing is set at a rate of 90 beats per minute for the first 7 days of the study. On day 8, the ICD is programmed to provide demand pacing at a rate of 40 beats per minute, which is the minimum rate possible with the device [16].

## 4. Pigs

Because of similarities of organ size, coronary anatomy, immunology, and physiology to humans, swine stand up as the most attractive model for pre-clinical protocols [31]. The three orders of magnitude difference in heart size between mice and humans and the abundance of collaterals in the dog coronary circulation make these experimental infarct models non-comparable to that of the human [32], but the swine and human myocardium share a high degree of similarity [9].

### 4.1. Blockage-Induced Myocardial Infarction (MI)

The procedure to induce MI in pigs by ligation is similar to the one described above for other animal models. In pigs, the LAD occlusion can be permanent [33] or temporary to allow for reperfusion. The reperfusion procedure is useful because, in current practice, most patients with acute MI receive reperfusion therapy [34]. In pigs, many different techniques for LAD occlusion are described.

#### 4.1.1. Balloon Catheter Procedure

Animal models of human disease should represent the human clinical disease as accurately as possible. In chronic post-infarction HF models, MI sizes have to be highly reproducible, with acute reduction in regional and global LV function. MI induced by balloon occlusion reflects chronic HF with moderate LV impairment, and is, therefore, an excellent model for chronic HF in humans [35]. A coronary balloon catheter is advanced over a guide wire and positioned in the proximal portion of the LAD coronary artery, below the origin of the first diagonal artery. To induce infarction, the LAD coronary artery is occluded by balloon inflation for the desired time [32,35,36,37]. For anti-arrhythmic medication, pigs are continuously infused with amiodarone throughout the procedure, beginning 15 min before the infarction [32].

#### 4.1.2. Collagen Injections

In general, balloon occlusion requires more personnel, laboratory time, and resources and the infarction has been more variable and sometimes less complete compared to the direct surgical LAD ligation. Microsphere embolization has been found to be less consistent, sometimes producing extensive infarctions into non-target regions, presumably through both the inability to visualize microsphere locations as well as reflux of the particle retrograde during the cardiac cycle. In general, compared to other methods (e.g., balloon- or microspheres-induced MI), collagen injection appears more efficient in terms of time, expense, mortality and has good reliability [31].

To induce MI, a vascular sheath is percutaneously inserted in the right femoral artery. A coronary guiding catheter is introduced and advanced to the ostium of the left main coronary artery, and contrast angiography is performed. A diagonal branch of the LAD is selected for embolization. A coronary artery guidewire is inserted into the diagonal artery of the LAD. An infusion catheter is advanced over the wire into the mid-portion of the artery and collagen suspension is gently injected to create MI. After the injection catheter is withdrawn, occlusion of the artery is confirmed by repeat angiography [31].

### 4.2. Tetralogy of Fallot (TOF)

TOF is a severe congenital heart disease characterized after the initial repair by the absence of a functioning pulmonary valve. This causes blood flow regurgitation in the RV during diastole, leading to progressive enlargement of the RV and pulmonary arteries. Valve replacement is, thus, often warranted at some point in the disease progression [38]. The RV overload needs to be mimicked in order to establish an easily reproducible porcine model of repaired TOF. After a left thoracotomy, a vascular clamp is longitudinally placed across the pulmonary valve annulus without obstructing the RV outflow tract. A pulmonary valve leaflet is excised, and the pulmonary infundibulum, annulus, and trunk are enlarged by a 2-cm–long elliptically shaped polytetrafluorethylene patch to ensure loss of valve integrity. Pulmonary artery banding, made of umbilical tape, is placed around the artery and secured for a final diameter of approximately 1 cm. This model shows a significant enlargement of the pulmonary annulus area and a marked pulmonary regurgitation. A pulmonary artery band is added to create mild pulmonary stenosis [39]. This model represents a reliable long-term swine model of RV dysfunction and dysynchrony, with echocardiographic measurements comparable to adult patients with early surgically repaired TOF [40].

## 5. Non-Human Primates

Non-human primate models have the advantage of significant physiological, metabolic, biochemical and genetic similarity to humans. Despite this advantage, there has been a relative paucity of non-human primate models used in the study of cardiac diseases that currently underlie the most common causes of morbidity and mortality, such as chronic myocardial ischemia leading to heart failure [41].

### 5.1. Balloon-Induced Myocardial Infarction (MI)

This model provides a heart size and rate comparable to the human. Under fluoroscopic guidance, a coronary catheter is used to engage the left main coronary artery. A guide wire and angioplasty balloon is passed into the mid-left anterior descending artery and the balloon inflated for 90 min. Myocardial infarction is confirmed by ST segment elevation on ECG and by subsequent serum assays for cardiac troponin and creatine kinase [42]. Clearly, the balloon-induced MI is a less invasive procedure compared to the open-chest surgery below [43].

### 5.2. Coronary Artery Ligation-Induced Myocardial Infarction (MI)

Coronary artery ligation-induced MI is another commonly-used procedure in non-human primates, but this procedure has to be conducted under open-chest surgery [44]. Briefly, the left anterior descending artery is subject to permanent or temporary (1–3 h) ligation followed by reperfusion (termed occlusion/reperfusion, or O/R). The ligation site could be varied along the artery so that varying sizes of myocardial infarction can be induced at the late state. Similar to other types of MI, ECG can be used to monitor the induction of MI and infarction size following ligation [44]. The major shortcomings of this procedure is the high mortality of animals due to arrhythmias, cardiac arrest, infection, and the open-chest-induced lung damage [43,45].

### 5.3. Tachycardia-Induced Congestive Heart Failure (CHF)

Although many animal models (e.g., mice or rats) of CHF have been demonstrated to be a useful model to investigate the underlying mechanism of CHF, nonhuman primates offer a unique opportunity to investigate the genetic aspects of this complex disease due to their close genetic and phenotypic similarity to the human [41]. In this model, a unipolar pacing lead will be placed to the posterior wall of the monkey left ventricle during open-chest surgery. Following a 2-week recovery, the animal heart will be pre-paced for 4 weeks and then be rapidly paced at 250 bpm for 3 weeks followed by 290 bpm pacing for 4 weeks to induce CHF. Echocardiography, hemodynamic, and ECG measurements are used to monitor heart failure, which demonstrates a persistent elevation in left ventricular end-diastolic pressure (LVEDP >15 mmHg), significant depression of +dP/dtmax, and development of visible edema or abdominal ascites [46].

## 6. Summary and Future Directions

The ideal animal model of cardiovascular disease should mimic the human subject metabolically and pathophysiologically, be large enough to permit physiological and metabolic studies, and develop end-stage disease comparable to that in humans. Given the complex nature of cardiovascular disease, no species or animal model will present an exact simulation of the human disease; therefore, models should be chosen according to the study aim. Large animal models are the closest models of human disease but they are not suitable for high throughput studies. Their advantages and disadvantages need to be considered when planning a study protocol using large animals (see Table 1).

For research aimed at clinical translation, it is imperative that initial results from rodent studies be confirmed in a large animal model more closely resembling the heart of humans. When choosing a large animal model, we need to consider the anatomical and functional differences between species. However, we must remember that these animal models are far from representing heart disease in humans. Most models involve the artificial generation of disease in what was previously healthy tissue, which differs greatly from the complex and progressive development of human cardiovascular disease. We must also consider economic and logistic disadvantages specific to the handling of each species. Table 2 covers the commonly-used large animal models for cardiac disease and whether the disease is artificially induced or spontaneous. This provides basic information about published models for each large animal. Spontaneous models are harder to generate in large animals, since they are less suitable than small animals for genetic modification and selection.

## Figures and Tables

**Table 1:** Advantages and disadvantages of large animal models.

Advantages	Disadvantages
Most physiologically and/or clinically relevant Allows for chronic studies to be undertaken Allows for cardiac function and responses to be assessed in the intact animal Responsive to all the techniques and measurements made in man Resembles more closely the human heart (young human heart is pig-like, older heart is dog-like)	Higher maintenance costs Harder to handle and house-specialized infrastructure and trained personnel needed Longer gestation time and lifespan Dog and pig: ischemia-reperfusion induced arrhythmias are more frequent Less suitable than small animals for genetic selection and production of transgenic strains and less spontaneous disease models

**Table 2:** Relevant large animal models for cardiac disease (only one reference is listed for each model. See the main text for more references).

Animals	Induced Model	Spontaneous Model
Dogs	MI [10]	ARVC [18]
	Non-ischemic HF [21]	DCM [8] DMD [22]
	SCD [17]	Valvular disease [23]
Sheep	MI [27]	n/a
	SCD [16]	
Pigs	Cryoinjury [47]	n/a
	MI [32]	
	TOF [40]	
Non-human Primates	MI [6] CHF [46]	n/a

ARVC: arrhythmogenic right ventricular cardiomyopathy; DCM: dilated cardiomyopathy; DMD: Duchenne muscular dystrophy; HF: heart failure; MI: myocardial infarction; SCD: *sudden cardiac death;* TOF: Tetralogy of Fallot.

## Abbreviations

The following abbreviations are used in this manuscript:

AF	atrial fibrillation
ARVC	arrhythmogenic right ventricular cardiomyopathy
AV	atrioventricular
DCM	dilated cardiomyopathy
DMD	Duchenne muscular dystrophy
GRMD	Golden Retriever muscular dystrophy
HF	heart failure
ICD	implantable cardioverter defibrillator
LAD	left anterior descending
LV	left ventricle
LVEF	left ventricular ejection fraction
MI	myocardial infarction
RA	right atrium
RV	rights ventricle
SCD	sudden cardiac death
TOF	Tetralogy of Fallot
TP	tachypacing
VT	ventricular tachycardia
WHHL-MI	Watanabe heritable hyperlipidemic myocardial infarction
